# Current Status and Issues for the Role of Occupational Health Physicians in Japan

**DOI:** 10.31662/jmaj.2018-0011

**Published:** 2018-09-28

**Authors:** Koji Mori

**Affiliations:** 1 Department of Occupational Health Practice and Management, Institute of Industrial Ecological Sciences, University of Occupational and Environmental Health, Kitakyushu, Japan

**Keywords:** Occupational Physician, Occupational Health, Duty, Training, Collaboration

## Abstract

This paper reviews the circumstances, current situation, and issues for Japanese occupational health physicians and discusses future developments. The Industrial Safety and Health Act requires workplaces that regularly employ 50 or more workers to appoint one occupational health physician. Their duties have been expanded by amendments to the Act, and they now have increased authority. Under these conditions, the occupational health physicians not only comply with laws and regulations but also follow a professional code of ethics. After the Act was amended in 1996, occupational health physicians had to complete additional training requirements. Basic training courses are provided by the Japan Medical Association (JMA) and the University of Occupational and Environmental Health, Japan. Most of the occupational health physicians certified by the JMA do not spend enough time on occupational health. Many Japanese occupational health work issues can be separated into issues of exposure, labor, and the occupational health system. Occupational health is characterized by changing needs because of new industrial structures and technological innovations. Given occupational health physicians’ expanding duties and professional development, they need to collaborate with one other and other occupational health staff to meet society’s expectations.

## Introduction

The Japanese occupational health system was developed based on the 1972 Industrial Safety and Health Act. Since then, we have faced new challenges, such as adverse health effects from handling hazardous materials, using visual display terminals and implementing information technology, death from overwork, increased mental illness, and an aging workforce ^(1)^. While occupational health support staff make important contributions, the Act relies on occupational health physicians’ expertise to ensure employees perform appropriate activities under the occupational health management system within the workplace. The Act has also responded to workers’ increased needs by gradually expanding occupational health physicians’ roles.

In this paper, I review the circumstances, current situation, and issues facing occupational health physicians and discuss future developments.

## Circumstances in the Role of Occupational Health Physicians in Japan

Occupational health physicians’ duties, authority levels, and training requirements are defined in occupational health related acts and ordinances established since the related ordinance of the Factory Act (Ordinance on Prevention and Hygiene against Factory Hazards) was revised in 1938 (Table 1). Currently, they are mainly prescribed by the Industrial Safety and Health Act and the Ordinance on Industry Safety and Health.

**Table 1.** Expanded Appointment Duties for Employers, and Roles, Authorities and Training Requirements for Occupational Health Physicians in Japan.

Year	Enactments and amendment of acts and ordinances	Appointment duty for employers	Roles	Authorities	Training requirements other than medical license
1938	Amendment of related ordinance of the of Factory Act	A factory physician at factories employing 500 or more workers	Maintaining hygiene Monthly workplace patrols Health checkups		
1940	Amendment of related ordinance of the of Factory Act	A factory physician at factories employing 100 or more workers			
1947	Enactment of Labor Standards Act	A hygiene manager with medical license at workplaces employing 50 or more workers	Managing hygienic practices in collaboration with non-physician hygiene manager		
1972	Enactment of Industrial Safety and Health Act and Ordinance on Industry Safety and Health	One occupational health physician at workplaces regularly employing 50 or more workers and two or more employing more than 3000 workers. Exclusive occupational health physician at workplaces regularly employing 1,000 or making 500 or more workers engaged in designated hazardous works.	Monthly workplace patrols Health checkups Other duties related to the health management of workers	Making recommendations to employers to fulfill their duties based on the Ordinance	
1988	Amendment of Industrial Safety and Health Act and the Ordinance		Monthly workplace patrols Health checkups and face to face guidance based on the results Work environment management Work management Other duties related to the health management of workers Health education Investigation of occupational illness causes and recurrence prevention		
1996	Amendment of Industrial Safety and Health Act and the Ordinance			Making recommendation to employers to fulfill their duties based on the Act Protect provision that employer shall not dismiss or otherwise retaliate against the occupational health physician because of health recommendations	Medical knowledge required to perform health care for work (main requirement is completion of basic training course)
2005	Amendment of Industrial Safety and Health Act and the Ordinance		Addition of face to face guidance for overworked workers		
2014	Amendment of Industrial Safety and Health Act and the Ordinance		Addition of face to face guidance based on stress checks		
2018	Amendment of Industrial Safety and Health Act and the Ordinance			Employers’ duty reporting the contents of the occupational health physicians’ recommendations to the health committee	

Under the related ordinance of the Factory Act, organizations employing 500 or more workers (revised to 100 workers in 1940) were required to appoint a factory physician to secure their workers’ health. Based on the 1947 Labor Standards Act, a hygiene manager with a medical license was required in workplaces with 50 or more workers ^(2)^.

These factory physicians, in collaboration with non-physician hygiene managers, were responsible for maintaining hygiene in the factory, mainly by making monthly workplace patrols and performing health checkups for workers.

In June 1972, following high economic growth in Japan, the Industrial Safety and Health Act was passed in response to the frequent occurrence of industrial accidents and various occupational diseases. In the 1972 Act, the doctor appointed to the workplace was named occupational health physician (*sangyoui* in Japanese). Workplaces regularly employing 50 or more workers were required to employ one occupational health physician under the Order for Enforcement of the Industrial Safety and Health Act. The Ordinance on Industrial Safety and Health required an exclusive occupational physician to be employed at workplaces which regularly employed 1,000 or more workers, or 500 or more workers who engaged in designated hazardous works. Workplaces employing more than 3,000 workers were required to employ two or more occupational health physicians. These obligations have not changed to date, although the need to appoint an occupational health physician at workplaces with fewer than 50 workers has been discussed.

## Expansion of Occupational Health Physicians’ Duties and Authority

While the appointed occupational health physicians may be exclusive or non-exclusive to a workplace, the Act requires both exclusive and non-exclusive physicians to fulfill the same duties. When the Industrial Safety and Health Act and its ordinances were enacted, occupational health physicians’ duties were limited to monthly workplace patrols, health checkups, and other work related to the health management of workers. In the 1988 amendment, however, their duties expanded to encompass health guidance based on health checkups, work environment management, work management, health education, and investigation of occupational illness causes and recurrence prevention. The 2005 amendment added a provision for face-to-face guidance for workers who worked long hours. In the 2014 amendment, stress checks were implemented in addition to face-to-face guidance based on stress check results and other measures ^(2)^. Thus, the amendments expanded occupational health physicians’ duties under the Industrial Health and Safety Act. In reality, Japanese occupational health physicians are reported to spend much more time on tasks other than health care compared to their European colleagues ^(3)^.

The occupational health physician is authorized to make recommendations to employers as part of their duties. In 1996, when that authority was upgraded from the Ordinance on Industrial Health and Safety to the Industrial Safety and Health Act, the protection provision was added that the employer shall not dismiss or otherwise retaliate against the occupational health physician because of health recommendations. Furthermore, the 2018 amendment of the Industrial Safety and Health Act required employers to report the contents of the occupational health physicians’ recommendations to the health committee.

In addition, some movements are pushing for using occupational health physicians outside the circumstances covered by the regulations. The working age population is rapidly shrinking in Japan; therefore, creating healthy older people who can work longer is an important challenge in maintaining social health. Thus, the current administration promotes health and productivity management in the workplace as a policy alongside initiatives such as recognizing excellent companies ^(4)^. In contrast to traditional risk management, health and productivity management includes investing health-promotion programs for employees to stimulate positive outcomes, such as improved productivity. These programs should be conducted under the top management’s leadership. Even under these programs, occupational health physicians are expected to serve as health experts within their companies.

The Industrial Safety and Health Act of Japan requires establishing an occupational safety and health management system for each workplace. However, if the top management’s awareness of occupational health and productivity management is improved and addressed as a management task, this policy will be implemented based on a unified company- or corporate group-wide policy. In addition, more companies are providing unified occupational health services to sites with fewer than 50 workers . Under these circumstances, the number of companies appointing occupational health physicians to supervise the occupational health of a company, or corporate teams outside the legal and regulatory framework, is also increasing gradually. These occupational health physicians are expected to communicate with management, plan company-wide occupational health programs, recruit and train occupational health staff, participate in performance appraisals, evaluations, or audits of the implementation status of occupational health activities at each workplace ^(5)^.

## Ethics for Occupational Health Physicians

Exclusively employed occupational health physicians sign employment contracts, while those who are nonexclusively employed have a quasi-delegate agreement with their employers. In this relationship, when occupational health physicians decide that the maintenance of their workers’ health is necessary, they are required to make recommendations to their employer based on their authority under the Industrial Safety and Health Act. According to these interests, occupational health physicians may experience various psychological pressures on their judgment and behavior; therefore, they not only comply with the laws and regulations, but also follow professional ethics specific to occupational health ^(6)^.

The code of ethics for Japanese occupational health professionals includes the *Code of Ethics for Occupational Health Physicians* prepared by the Institute of Health Development Science in 1998 ^(7)^ and the *Code of Ethics Code for Occupational Health Professionals* published by the Japan Society for Occupational Health (JSOH) in 2000 ^(8)^. The International Labor Office’s *Technical and Ethical Guidelines for Workers’ Health Surveillance* ^(9)^ and the International Commission on Occupational Health’s *The International Code of Ethics for Occupational Health Professionals* ^(10)^ are also available.

The occupational health physician is required to behave ethically in a variety of situations and demands; thus, they may experience difficulties in acting ethically simply by following an ethical code. They must make continuous efforts to act as independent professionals working under laws and ethics in the situations that may arise at their workplace. These efforts include learning through experiences and discussions with other occupational health physicians. Few organizations employ more than one occupational health physician; therefore, they seek various opportunities for discussing ethical behavior by participating in academic societies and research groups, among other avenues.

## Training and Qualifications of Occupational Health Physicians

The factory doctor in the Factory Act era, the hygiene manager with a medical license in the Labor Standards Act era, and the occupational health physician at the time of the establishment of the Occupational Safety and Health Act were required only to be doctors ^(2)^. However, as mentioned above, occupational health physicians’ duties have expanded, and so must their knowledge base. The 1996 amendment to the Occupational Safety and Health Act, which enhanced occupational health physicians’ mandated that they satisfy at least one of the requirements specified by the Ordinance of the Ministry of Health, Labor and Welfare (MHLW) on medical knowledge required to perform health care for workers. The main requirement is to complete training conducted by entities designated by the MHLW, which are currently the Japan Medical Association (JMA) and the University of Occupational and Environmental Health, Japan (UOEH). The major training courses conducted by JMA and UOEH are JMA’s Basic Training Course on Occupational Medicine ^(11)^ and the UOEH’s Occupational Medicine Intensive Course ^(12)^. Both of these courses are worth 50 credit hours.

JMA’s Basic Training Course on Occupational Medicine has been provided since February 1990, before the Industrial Safety and Health Act requirements were established ^(11)^. As of January 30, 2018, 97,562 doctors met the minimum training requirements and were certified by JMA. It is not a legal requirement, but more than 20 hours of training is required over 5 years as a contribution to their lifelong training to maintain their certificate. After analyzing the themes of the training courses in 2006 and 2007, 4,822 and 6,750 training sessions were held nationwide, respectively. The theme selection and number of offers appeared to be sufficient ^(13)^. However, longer practical training is necessary to fulfill the expanded duties for occupational health physicians as required by the regulations. Specifically, to ensure that the duties of the occupational health physicians were fulfilled, an estimated 126 hours of practical training was needed in addition to the 50 hours of the basic training course ^(14), (15)^.

Most JMA-certified occupational health physicians work as clinicians and spend only part of their time as occupational health physicians. However, the number of physicians whose main duties cover occupational health is gradually but steadily increasing. In Europe and the United States, specialist occupational health physicians complete their comprehensive training in Master of Public Health educational programs ^(16)^. In Japan, however, there are extremely limited opportunities to access the comprehensive programs, except for the postgraduate courses for UOEH graduates. While on-the-job training is the focus, the JSOH operates a system for training and certifying professional occupational health physicians ^(17)^. Under this system, a doctor aiming to become a JSOH-certified professional occupational health physician received on-the-job training, supervised by a qualified senior occupational health physician, for three or more years and then passed a final qualification examination. The level of the required competency is almost the same as for specialist occupational health physicians in Europe and the United States ^(18)^. As of April 1, 2018, 592 occupational health physicians have been certified since the system was established in 1993.

## Occupational Health Issues in Japan

In Japan, occupational health issues can be divided into exposure issues, such as the workplace environment and style, labor issues, and occupational health system issues.

Occupational diseases directly attributable to work have decreased compared to an era of high growth with the occurrence of various diseases. However, back pain due to manual handling of heavy objects or bad posture, heat illness during outdoor work in summer or in-house work in high temperatures, and high humidity are still important issues ^(19)^. There were detailed management methods for regulating high-risk chemical substances, but recent concerns have grown about unregulated substances leading to cases of cholangiocarcinoma due to dichloropropane exposure ^(20)^ and of bladder cancer due to ortho-toluidine ^(21)^. The MHLW obliges risk assessment of chemical substances and requests employers to manage them voluntarily according to the results.

Measures to prevent adverse health effects from psychosocial factors, such as working long hours and stress, have been enhanced in recent years. However, the number of cases compensated by labor accident insurance remains high. The government proposes reducing working hours in one of its major themes of working-style reforms ^(22)^.

Occupational health is characterized by the factor that needs are changing because of new industrial structures and technological innovations. For example, technological innovations represented by the Internet of things and artificial intelligence are creating new industries; therefore, major changes in the working environment and styles are expected. Among these changes, occupational health physicians pay attention to changes in the risk of work-related adverse health effects. They also make efforts to utilize new technologies in the occupational health field.

Occupational health also faces major challenges in labor issues as well as working environment and style. The aging workforce creates situations where workers may have various age-related health conditions, including lifestyle diseases, but are still working. Thus, efforts to maintain and promote health are more important than ever. It is also necessary to support workers who are suffering from diseases, such as cancer, and enable them to both continue working and receive treatment. These efforts were promoted as part of the working-style reforms described above ^(22)^.

There is a serious change in the labor force because of Japan’s increasingly aged population. As a countermeasure, various industries are increasingly employing foreign workers. Thus, these workers’ health problems may be different from Japanese workers, and it is necessary to ensure their health care. In addition, occupational health education is necessary to prevent health disorders due to work-related factors.

Finally, considering issues related to the occupational health system, the appointment rate of occupational health physicians is low in workplaces with fewer than 50 employees where it is not obligatory to contract with an occupational health physician. In these workplaces, the only occupational health services provided are medical checkups. The MHLW has been expanding its support through its recently established Regional Occupational Health Center, among other facilities ^(23)^. These services are not widely used; therefore, from the workers’ perspective, there is a big difference in occupational health services that can be received depending on workplace size.

Most physicians worked fewer than 6 hours per month ^(24)^. Considering their expanding duties, such as monthly workplace patrols, health committee attendance, providing necessary advice on the results of health checkups, and providing face-to-face guidance for workers who worked long hours, it is difficult to say that the time available for service is sufficient. Furthermore, occupational health physicians’ quality improvement is not keeping pace with the expansion of their duties, and the locations of occupational health physicians are not distributed evenly ^(19)^.

## Collaboration Among Occupational Health Physicians and with Other Occupations

The Industrial Safety and Health Act of Japan requires occupational health physicians to acquire and maintain expert knowledge needed for performing health-care measures. However, it is becoming difficult for occupational health physicians to complete their expanding duties following their basic training. More than 160,000 workplaces are obliged to employ an occupational health physician; thus, approximately 2,000 to 2,500 exclusively employed occupational health physicians are required ^(25)^. Obviously, these needs cannot be met by only specialist occupational health physicians who have had comprehensive training in a wide range of occupational health themes and who are constantly studying at academic societies, such as JSOH. Therefore, under these circumstances, collaboration among occupational health physicians or with other occupational health staff is essential. Most of the doctors appointed as occupational health physicians have difficulty fulfilling all the duties. A team approach for occupational health physicians was previously discussed by a MHLW committee ^(26)^. In this approach, a group of occupational health physicians were assigned to a workplace. Among them, one was designated as the supervisor who coordinated all the physicians’ tasks, and they collaborated with each other to provide high-quality services by sharing information. However, this approach was not realized because of some concerning factors. Nevertheless, in response to an increase in mental illness, several workplaces have employed a physician specializing in mental health measures in addition to the occupational health physician.

The Industrial Safety and Health Act and its related regulations stipulate that working environment measurement experts conduct working environment measurements, public health nurses provide health guidance, and public health nurses, nurses, and psychological social workers conduct stress checks. Although these provisions address specific tasks of occupational health services, in reality, occupational health staff collaborate with each other on a broader range of occupational health services. The related MHLW committees also discussed this type of collaboration ^(27), (28)^. There are a variety of actual cases of collaboration between occupational health staff members depending on the company conditions, which could be used to produce a best practice model.

In addition, improving other occupational health professionals’ skills is a prerequisite for occupational health staff collaborations. In 2015, JSOH began a training and certification system for public health nurses and nurses engaged in occupational health services ^(29)^. Managing workplaces where hazardous materials are used is difficult raises legal challenges, such as working environment measurements. Thus, trained experts are necessary for voluntary management of hazardous materials. In Europe and the United States, these experts are called occupational or industrial hygienists. In Japan, however, there are few such experts, even though the Japan Association for Working Environmental Measurement has provided an occupational hygienists training system since 2007 ^(30)^. Future developments are expected.

More than one-fourth of doctors have completed the basic training necessary to perform health care for workers in Japan. This knowledge should be used in preventive efforts, even in the clinical field. In addition, clinical doctors in medical institutions and occupational health physicians in the workplace should collaborate in enabling workers suffering from diseases to be able to both continue working and receive treatment. If the doctors in the medical institutions are experienced occupational health physicians, to the collaboration will be relatively smooth.

## Conclusion

Occupational health physicians are expected to resolve workers’ health issues; thus, their duties have expanded in both legal and practical terms. In addition, occupational health physicians are in a difficult position as they must make their recommendations based on the Industrial Safety and Health Act while employed in a workplace. Thus, special knowledge of occupational ethics is required. Under these circumstances, occupational health physicians may have limited knowledge if they have only completed the minimum training requirements required under the Industrial Safety and Health Act. Therefore, it is necessary to establish a framework to ensure that occupational health physicians can contribute to the protection of workers’ health and secure healthy workforces by collaborating with other occupational health physicians and other staff members who have received comprehensive training.

## Article Information

### Conflicts of Interest

None

### Sources of Funding

This work was supported by the Industrial Disease Clinical Research Grants from the Ministry of Health, Labour and Welfare, Government of Japan (170302-01).

### Acknowledgement

We thank Peter Fogarty, MA English 1st Class, from Edanz Group (www.edanzediting.com/ac), for editing the English text of a draft of this manuscript.

### Author Contributions

KM performed the process and developed the paper.
